# Ethical Issues and Challenges Regarding the Use of Mental Health Questionnaires in Public Health Nutrition Research

**DOI:** 10.3390/nu17040715

**Published:** 2025-02-18

**Authors:** Karim Khaled, Fotini Tsofliou, Vanora Hundley

**Affiliations:** 1 Department of Public Health, Faculty of Health, Education, & Life Sciences, Birmingham City University, Edgbaston, Birmingham B15 3TN, UK; 2 Department of Rehabilitation & Sport Sciences, Faculty of Health & Social Sciences, Bournemouth University, Bournemouth BH8 8GP, UK; ftsofliou@bournemouth.ac.uk; 3 Centre for Wellbeing and Long-Term Health, Faculty of Health & Social Sciences, Bournemouth University, Bournemouth BH8 8GP, UK; 4 Centre for Midwifery and Women’s Health, Faculty of Health & Social Sciences, Bournemouth University, Bournemouth BH8 8GP, UK; vhundley@bournemouth.ac.uk

**Keywords:** mental health questionnaires, desk-based research, ethical concerns, ethical challenges, ethical issues, public health nutrition, epidemiological research, ethics, nutrition, depression, depression scale

## Abstract

Background: The use of mental health questionnaires is common in desk-based public health epidemiological research; however, the burden this might put on participants and researchers has been questioned and has not been previously addressed. This paper delves into the ethical issues and challenges of using such scales and questionnaires, providing a real-life case study where the Beck’s Depression Inventory-II was used. Methods/Results: The ethical considerations raised by using mental health questionnaires in public health epidemiological research include incorrectly identifying participants as depressed or non-depressed; inability to identify participants for referral procedures due to the anonymous nature of some research studies; an increased burden on participants through depression and suicidal questions; and the high expectation of participants towards the researcher. Preventative measures to reduce these challenges include choosing appropriate cut-off scores for correctly identifying participants; highlighting whether the mental health questionnaires used may elicit negative emotional or psychological reactions related to suicidality; specifying the criteria for referral to clinical services; detailing the intended referral processes; including approaches where the researcher directly connects participants with a psychological service provider; and including a passive referral method such as contact details for participants to initiate their own referrals to clinical care. Conclusions: This paper serves as a guide for researchers aiming to collect data on mental health through questionnaires. The ethical challenges discussed in this paper should be considered and reviewed at all stages of the research project.

## 1. Background

Diet, as a modifiable lifestyle factor, has been closely associated with non-communicable diseases (NCDs) such as cardiovascular diseases, diabetes, cancer, and obesity [1]. More recently, there has been a focus on mental health disorders and their impact on diet, and vice versa [2]. Research indicates that depression serves as a significant risk factor for mortality, with its impact being comparable to that of smoking [3]. The Global Burden of Disease (GBD) study, which assesses the health implications of over 100 diseases and injuries, revealed that in 2019, depression was the foremost cause of disability among middle- and high-income populations [4]. Research in nutrition provides valuable insights into the relationship between dietary patterns and health outcomes, guiding public health policies and interventions [5]. By understanding the nutritional needs of diverse populations, researchers can identify risk factors, develop targeted nutritional guidelines, and promote health equity [6]. Ultimately, nutrition-focused public health research is key to reducing the burden of preventable diseases and improving the quality of life across communities [5,6].

Nonetheless, this field of research is fraught with challenges. The predominant methodologies employed in dietary assessment of nutritional surveys include diet records, 24-h recalls (24HRs), and food frequency questionnaires (FFQs) [7]. Each of these approaches presents its own set of advantages and limitations. It is important to note that all dietary assessment methods are susceptible to both random and systematic measurement errors [8]. Despite the inherent measurement errors associated with dietary assessment tools, they remain valuable for research, monitoring, and policy development as they enable healthcare professionals to methodically evaluate the nutritional condition of patients, diagnose instances of malnutrition, uncover underlying health issues contributing to malnutrition, and propose appropriate intervention strategies [8]. A comprehensive understanding of the various obstacles within public health nutrition research necessitates the exploration of several critical components such as research methodologies, research participants, and strategies for gathering and analysing information [9]. However, the ethical challenges that can arise from using desk-based research tools are rarely reported.

While the links between nutrition and mental health are becoming increasingly recognised, research in this interdisciplinary field presents a set of unique challenges, particularly from an ethical standpoint. The integration of mental health assessment tools, such as depression screening scales, into public health nutrition research raises several considerations that are distinct from traditional nutrition research. Unlike typical dietary assessments, which focus primarily on physical health and nutritional status, mental health questionnaires often involve deeply personal and sensitive information [10]. These tools are designed to screen for conditions that can significantly impact a participant’s well-being, yet they are typically employed in a research context where clinical interventions are not provided [9]. This gap between research and clinical care creates a dilemma, as participants may not be fully prepared for or informed about the potential consequences of a positive mental health screen [8]. Furthermore, the use of such questionnaires in populations that may not be seeking clinical help may inadvertently raise concerns about stigmatisation or misdiagnosis [9]. These factors make the ethical challenges in nutrition-related mental health research particularly pressing, as they require careful consideration of participant rights, the role of researchers in identifying mental health conditions, and the potential psychological impact of such screenings.

Several validated health and well-being-related questionnaire tools are used in desk-based research, where the researchers are looking at associations of chronic disease with lifestyle factors, demographics, socioeconomic, and physical factors rather than using the tool to screen and treat a condition [10]. Examples of commonly used tools in desk-based research include those used to measure quality of life (e.g., Activity of Daily Living Scale), anxiety (e.g., State Trait Anxiety Inventory), and depression (e.g., Beck Depression Inventory and the Hospital Anxiety and Depression Scale) [10]. These instruments have been validated across various countries and diverse populations which make them commonly used in desk-based research [11]. Their straightforward design, presented in a familiar standardised test format, provides clear and formal instructions for researchers while allowing participants to easily respond to the questions [11]. For instance, epidemiological and public health nutrition researchers examining the links between health, diet, and mental health symptoms have employed these tools in their methodologies as a key measure of depression [12].

Questions have been asked about the ethics of using such scales in non-clinical research; these include questions of whether participants are aware of the implications of a positive result and whether the researcher should refer participants that score highly [13]. It can be contended that neglecting the possibility of depression may raise ethical concerns [13]. When participants in a research study receive a positive screening result for depression based on the established cut-off score of the assessment tool, the subsequent management protocol for their care remains ambiguous. This paper explores the ethical issues and challenges regarding the use of mental health questionnaires in public health nutrition research, providing a real-life case study in which one of the mental health questionnaires (Beck Depression Inventory-II) was used in a PhD project.

## 2. Ethical Issues and Challenges Raised by Using Mental Health Questionnaires in Public Health Nutrition Epidemiological Studies and Suggestions to Reduce Them

In public health nutrition research, mental health questionnaires are frequently used without considering potential ethical issues or challenges. This section discusses these challenges and the strategies that can be implemented to mitigate them (Table 1) [13].

The Beck Depression Inventory II (BDI–II) is among the most frequently utilised tools for depression screening, favoured for its brevity, user-friendliness, and proven reliability and validity [14,15,16]. The BDI–II has been translated into numerous languages and validated across diverse ethnic groups, including clinical and non-clinical populations, adolescents, adults, elderly, individuals with substance use disorders, and those with intellectual disabilities [14]. A review by McPherson et al. [15], which analysed data from 34 validation studies of the BDI–II, revealed high internal consistency scores ranging from 0.82 to 0.96, strong test–retest correlations between 0.7 and 0.9, and a consistently stable unidimensional structure across various studies.

**Table 1:** Key ethical issues and challenges that might arise from using mental health questionnaires and mitigation strategies to reduce them.

Ethical Issue	Potential Risks	Mitigation Strategy
**Informed Consent** [16]	Participants may not fully understand risks or voluntary nature of participation.	Provide clear, concise consent forms outlining study purpose, potential emotional distress, and referral protocols.
**Emotional Distress from Mental Health Questions** [17]	Increased anxiety, depression, or suicidal ideation.	Include a disclaimer about potential distress in the consent form, with specific instructions on how participants can withdraw.
**Confidentiality of Sensitive Data** [17,18]	Breach of confidentiality regarding mental health or dietary data.	Ensure data anonymization and secure data storage. Implement clear policies for data access and sharing.
**False Positives/Negatives in Depression Screening** [17]	Incorrectly identifying participants as depressed or not.	Use validated mental health screening tools. Include a disclaimer about potential false results and offer a referral for re-assessment.
**Inability to Follow-Up with Participants for Mental Health Referrals** [18]	Failure to connect participants with appropriate mental health services.	Implement a “passive referral” method (e.g., provide contact details for mental health services) and/or a follow-up procedure.
**Psychological Burden on Participants** [19]	The emotional toll of answering distressing questions may deter participation.	Pre-screen participants, provide them with resources to support them emotionally, and assure them of their right to skip questions.
**High Participant Expectations of Researchers** [17,20]	Participants may expect researchers to provide professional mental health support.	Make clear in the consent form that researchers are not trained mental health professionals and that referral services will be offered.
**Researcher Guilt or Emotional Burden** [20]	Researchers may feel responsible for participants’ emotional distress.	Offer researchers ethical training on dealing with emotional distress in participants and provide support for researcher well-being.

Questionnaires such as the BDI-II are frequently used in public health nutrition research without serious consideration of their potential effects on participants and researchers [16]. Potential psychological impact could arise from questions within these questionnaires that touch on sensitive psychological topics without serious consideration of their potential effects on participants and researchers.

There is a scarcity of evidence to either confirm or refute the idea that inquiries related to mental health questionnaires (particularly those addressing issues like depression, anxiety, or suicide) may cause harm to study participants. It is likely that participants in any population-based research study might have pre-existing latent mental health conditions or may have recently endured traumatic experiences, such as the loss of a loved one [10]. These factors can render participation in research employing mental health questionnaires a delicate issue for many participants [12]. These ethical issues will be considered below along with the preventative measures that researchers can adopt. A comparative analysis of the challenges, and a summary of the measures to address these, are included in Supplementary File S1.

### 2.1. Psychological Burden

#### 2.1.1. Incorrectly Identifying Participants as Depressed or Non-Depressed

An ethical challenge to consider in desk-based research is the potential for misidentifying participants as depressed, commonly referred to as “false positives” [21]. That is, achieving the cut-off scores for depression on screening tools does not necessarily confirm a clinical diagnosis of depression [21]. Research indicates that approximately 59% of individuals who screen positive for depression in desk-based research are misclassified, resulting in false-positive outcomes [22]. This can lead to research participants being wrongly labelled as depressed and receiving unsuitable treatment in certain studies [23]. Consequently, researchers should be responsible to critically evaluate the psychometric characteristics of the selected mental health screening tool, which typically differ based on patient demographics (including gender), age, and the specific type of depression questionnaire being used [24]. Utilising a cut-off score with low specificity is a key factor that puts researchers at risk of mislabelling study participants who might not necessarily require medical and mental health referral as depressed [25]. Therefore, it is essential that desk-based researchers investigate the sensitivity and specificity of the mental health screening tool that they intend to use (e.g., BDI-II) within their specific study population to allow for an appropriate and accurate selection of cut-off diagnosing scores [24]. Researchers should be aware that data regarding the diagnostic accuracy of screening tools in specific populations are not always easily accessible, highlighting the need for further investigation into optimal cut-off scores [25]. However, a high specificity threshold of 95% or greater is advisable to reduce the risk of false positives. Nonetheless, the U.S. Preventive Services Task Force (USPSTF) states that it is the responsibility of researchers conducting mental health screenings (e.g., depression) to ensure accurate diagnosis, effective treatment, and appropriate follow-up care for depression [13].

#### 2.1.2. Inability to Identify Participants for Referral Procedures Emerging from the Anonymous Nature of Some Research Studies

Several systematic reviews stated that communicating screening outcomes to primary care providers can lead to enhancements in depressive symptoms, particularly when supplementary personnel are available to offer support for depression care [26,27]. However, no significant benefits were observed in a systematic review of scenarios lacking collaborative care or systemic enhancements, which include research training, the distribution of educational resources for participants, the presence of support staff, follow-ups, and referrals to mental health services [28]. Within this context, researchers should be aware that the partnership with mental health professionals is crucial to ensure effective management, thereby enhancing both diagnostic precision and the overall quality of care [29]. In instances where positive screening results are to be communicated to medical professionals, it is essential that participants are fully informed and consent to this referral process, in alignment with ethical standards [30]. The participant information leaflet and consent form must explicitly convey this information [30]. While the results of depression screenings could remain confidential, participants must be made aware that such results will be shared with their medical team if a positive screen occurs [13]. Furthermore, they should be informed about which specific members of the medical team will have access to this information and retain the right to refuse consent for its disclosure [29]. Husain et al. [31] emphasised that at a minimum, researchers should inform participants identified as “at risk” about the necessity for further evaluation for depression or provide general written information regarding depression to all participants. Nonetheless, access to information regarding the care options available to research participants is not consistently guaranteed, a concern that is especially relevant in studies conducted across multiple locations [31]. Furthermore, the act of reporting positive screenings raises significant concerns regarding confidentiality and the principles of informed consent [17]. This might be more challenging when the participation in research is anonymous [17]. However, the researcher can include a statement regarding the potential for triggering and links to people who can help [18]. An example consent form and referral protocol are given in Supplementary File S2.

Desk-based researchers often use anonymous surveys that are administered online to explore factors associated with health and nutrition [32]. These anonymous surveys are designed in a way that refrain from identifying participants and do not gather personally identifiable data (e.g., names, email addresses, phone numbers, social media accounts, and identification numbers) [32]. Participants will more likely be encouraged to take part of research in which their identifies are not identifiable and where they can provide more honest responses to sensitive topics without being embarrassed [33]. This is to say that not only the response rate increases through anonymity, but also the accuracy of feedback to questions within the surveys [34]. When participants have the opportunity to respond to a survey without revealing their identities, they will have no reason to provide false information, thereby enhancing the overall reliability of the research findings [34]. Despite these benefits, the ethical considerations surrounding anonymity and confidentiality are paramount, especially in research assessing mental health, where populations or individuals who have serious depressive symptoms, suicidal thoughts, or behaviours may be included [35]. It is thus recommended that researchers provide potential participants with clear information regarding the focus of the surveys, utilising a scripted approach or passive consent for participation depending on the nature of research participation (in person or online) [36]. In case of in-person anonymous participation, it is essential to offer participants with guidance on accessing local resources for additional counselling or support if they feel the need after completing the surveys [37]. For example, investigators should provide national hotline numbers alongside local suicide prevention resources for further assistance or information after completing the surveys [38]. Similarly, in the case of online participation, researchers should compile a list of nationally accessible suicide prevention resources or helplines for participants seeking further information post-survey completion [38]. Further details are discussed below.

In every circumstance, an additional stipulation must be acknowledged. All participants are required to engage in the study with a clear understanding of its implications [39]. It is particularly important to inform individuals who are contemplating participation that they may encounter inquiries that could be particularly distressing, especially since, under the updated study protocol, they will be required to complete the questionnaire after their diagnosis has been disclosed [39]. It is evident that participants cannot completely anticipate this scenario at the initial recruitment stage [35]. Nonetheless, they should be encouraged to reflect on their potential feelings regarding the promotion of informed consent [36]. The consent stage serves as a critical juncture for addressing the ethical issues identified in Table 1 [20]. Additionally, it provides an opportunity to mitigate another issue, which is drawing unrealistic and inappropriate expectations [20]. Thus, it has been recommended by ethics committees to incorporate a statement at the beginning of each questionnaire, indicating that participants are not required to respond to any questions they find distressing or inappropriate [40]. Additionally, ethically responsible researchers should always remind participants throughout the questionnaire that they have the right to withdraw from the study at any point without the need to provide any justification [40]. Nevertheless, questionnaire design typically aims to encourage respondents to engage comprehensively with each item to improve the validity of the findings [41]. Thus, a careful balance must be achieved, necessitating a thorough examination of the study design to evaluate the effects of any non-completion of questionnaire items [41].

#### 2.1.3. Risk of Suicide

Some of the items commonly found in mental health questionnaires address suicidal thoughts, which present serious ethical challenges for researchers [17]. According to both ethical standards and clinical guidelines outlined in the depression screening manuals, individuals who indicate suicidal ideation must receive appropriate follow-up [17]. In research, it is not standard practice for researchers to disclose the methods employed to address positive screening results identified during a study [18]. Increasingly, research ethics committees have been recommending that researchers implement a response strategy for participants with elevated depression screening scores prior to the initiation of recruitment and data collection [19]. Additionally, recommendations from the American Heart Association (AHA), the U.S. Preventive Services Task Force (USPSTF), and the National Institute for Health and Clinical Excellence (NICE) all endorse that individuals who screen positively for depression should be considered for referral to a qualified professional for a thorough evaluation and management of their condition [42].

It can be argued that researchers employing these depression screening questionnaires (e.g., BDI-II) should incorporate a systematic approach within their protocols to manage any positive responses related to the scale’s items on pessimism and suicidal thoughts [43]. The United States Preventive Services Task Force (USPSTF) advocates for clinical research that implements depression screening to establish systems that facilitate accurate diagnosis, effective treatment, and appropriate follow-up care [44]. The optimal strategy for addressing cases where participants exhibit positive suicidal and depression diagnosis in research involves the documentation of these positive results within a framework of collaborative care [44].

#### 2.1.4. Impact on Study Researchers—Expectations and Feelings of Guilt

Another ethical challenge to consider in this paper is with regard to study researchers [20]. Although the foremost concerns should be viewed as those related to the interests and welfare of the research participants, the potential risks to the researcher also hold ethical significance and warrant attention [45]. In this context, the risks to researchers are linked to the expectations that some participants may have of them [45]. It poses a challenge when a researcher is erroneously perceived as a provider of assistance that they are unable to offer, especially given the serious and distressing conditions that occupy the thoughts of these patients [45]. It is reasonable to anticipate that this situation could evoke a sense of guilt in the researcher, even if such feelings are unwarranted [46]. Therefore, researchers are advised to make clear statements in the consent form, participants information sheets, and in the surveys of what their exact responsibilities and duties are and what participants should expect from them [46]. This can taper down or wipe away the feeling of guilt that some researchers experience, especially when proper referral procedures are there to be implemented [46].

## 3. Case Study

In my PhD, I (K.K.) aimed to assess the association between dietary patterns in women of reproductive age and their psychological status (stress and depression) in three countries (UK, USA, and Lebanon) through an online survey. This was an observational, cross-sectional study and the papers are reported in the full thesis [47]. After appraising the literature about the topic, I found that many nutrition epidemiological studies used the Becks Depression Inventory-II as a measure of the depression and psychological status of participants. Thus, I decided, with my supervisory team (F.T. and V.H.), to include the scale in my study, which employed an anonymous survey to collect the data.

Before launching the survey, I completed the university’s ethics checklist and sought approval in order to start recruitment. I was surprised that when I presented my research in front of the university’s ethics committee, many questions were raised regarding the use of Beck’s Depression Inventory in my study. The ethics committee felt that it was unethical to use the scale without appropriate follow-up. The committee questioned how the researcher would deal with the situation where a participant scored highly and required medical input.

A further concern was that the student (K.K.) and primary supervisor (F.T.) came from a nutrition background and were not mental health professionals. Although the study was intended to be desk-based research and there was no intention to detect clinical depression, the ethics committee noted that the team did not have the skills to identify and refer participants if they required medical input.

### 3.1. Intended Use of the Scale

I welcomed the opportunity to review the literature and debate the challenges raised by positive screening scores. The BDI-II is commonly used to provide information about depressive symptoms as part of the research [48]. In such situations, the intention is to identify lifestyle factors associated with higher scores rather than identify individuals who require clinical treatment [44]. A further, but important, consideration is that screening scores are not diagnostic for depression, and in all cases, a clinical examination is required [18]. Therefore, I argued that simply having a self-reported positive depression score in my survey would not be a diagnosis for depression, especially since I have excluded participants with mental illness and included only healthy women of reproductive age.

### 3.2. Feasibility

Since screening and referral were not the intention of my research, the results would not be returned to participants. The majority of questionnaires were to be completed anonymously and therefore follow-up would not be possible.

As discussed above, it is advised that researchers provide information about community resources to help participants [18,19]. Therefore, I added a link to resources following the Beck Depression Inventory II questionnaire and at the end of the online survey. These community resources (e.g., the Samaritans, Bournemouth) were added for all participants who wished to seek further support. In addition, the survey contained the telephone numbers and email addresses of the student well-being service at the university.

The panel were also concerned that the BDI-II contains physical symptom items such as changes in appetite or fatigue that may be related to an illness or treatment such as congestive heart failure or chemotherapy and may not reflect a change indicative of depression, leading to overestimating the occurrence of depression [20,42]. This was acknowledged, but the PhD project was targeting women of reproductive age who were healthy (participants with any chronic or mental diseases were excluded), and therefore I was able to argue that this overestimation would be unlikely to occur.

This case study provides valuable insights into the ethical challenges encountered in a public health nutrition study involving mental health questionnaires, particularly those addressing suicidality. The decisions from the ethics committee included the careful inclusion of sensitive mental health questions, accompanied by precautionary measures such as informed consent and access to mental health resources. A passive referral system was implemented to respect participant autonomy while ensuring access to support services. Alternative approaches, such as excluding suicidality questions or adopting an active referral system, were considered but rejected due to concerns over data limitations and participant anonymity. Feedback from the ethics committee led to the inclusion of validation checks for mental health assessments and clearer trigger warnings to mitigate psychological burden. The lessons learned emphasise the importance of transparency, flexibility, and ongoing ethical reflection, ensuring that ethical concerns were continually addressed throughout the study’s design and stages. This process highlights the need for proactive ethical decision-making in future public health nutrition and mental health research.

## 4. Practical Implications in Relation to Research on Human Nutrition

### 4.1. Training Regarding Education of Students in Nutrition

Addressing the ethical issues in nutrition research has significant practical implications, particularly in the realm of training students in nutrition education. Ensuring that future nutrition professionals are well-versed in ethical principles—such as informed consent, confidentiality, and responsible data interpretation—prepares them to navigate complex research landscapes with integrity [49]. Incorporating ethical considerations into curricula helps students recognise the importance of participants’ rights, potential risks to participants and researchers, referral procedures in certain cases, equity in research design, and the potential biases in studies [50]. Furthermore, it cultivates a deep understanding of how to ethically communicate findings to both the scientific community and the public, ultimately fostering trust in nutrition science [51]. By integrating ethical training into the educational framework, institutions can produce nutrition experts who are not only scientifically proficient but also committed to upholding the highest ethical standards in both research and practice [52]. This has long-term implications for the credibility and reliability of nutrition science as it continues to evolve [52].

### 4.2. Links Between Academia and Clinical Practice

Addressing the ethical issues in nutrition research also has critical implications for bridging the gap between academia and clinical practice, ensuring that research findings are translated into effective, evidence-based clinical interventions [53]. Ethical considerations such as transparency in study design, data integrity, and the responsible application of research outcomes are vital in maintaining the trust and collaboration between researchers, clinicians, and patients [54]. When academic nutrition researchers are aware of ethical issues and their preventative measures, they adhere to strict ethical standards which are applicable in real-world settings, fostering a smoother transition from research to practice [55]. This is particularly important when translating dietary guidelines or mental health interventions that directly impact patient care [55]. By addressing ethical challenges, both academia and clinical practitioners can work together to ensure that nutrition recommendations are based on unbiased, high-quality evidence, which ultimately enhances patient outcomes [56]. Furthermore, ethical training in nutrition research prepares future professionals to navigate the complexities of real-world clinical environments, where patient autonomy, informed consent, and culturally sensitive interventions are key [57]. This ethical framework ensures that nutrition research not only informs clinical practice but also aligns with the broader goals of patient well-being and public health. Future work should consider how roles such as clinical academics can be developed to support joint working between academia and practice and ensure mutual understanding.

### 4.3. Funding and Governance

Both funding sources and governance structures can profoundly shape the scope, integrity, and societal relevance of nutrition research [58]. Ethical funding and governance are critical in nutrition studies, as they often involve human participants, where issues such as informed consent, privacy, and the handling of sensitive dietary and mental health information must be addressed [59]. They also ensure that studies adhere to national and international standards, particularly concerning food safety, mental health, and public health regulations [59]. In the context of nutrition research, governance mechanisms can include specific guidelines on how studies should account for population diversity, considering variations in diet, culture, socioeconomic status, and mental health conditions [60]. For example, research examining nutritional interventions in vulnerable populations (e.g., children, pregnant women, or people with chronic conditions) requires particularly stringent oversight to ensure that the risks to participants are minimised and the benefits are maximised [61].

## 5. Conclusions

The insights derived from this paper can be categorised into two primary conclusions. Firstly, there is a critical necessity for comprehensive consent protocols that proactively address and mitigate the potential risks faced by all involved parties (participants and research investigators) [7,8]. Secondly, conducting epidemiological and nutritional questionnaire-based research that includes mental health questions could elicit psychological effects such as increased anxiety, depression, and/or suicidal levels, and this needs to be carefully considered by researchers [10,62].

This is not to say that researchers should refrain from the critical endeavour of exploring health-related behavioural or psychological issues in public health nutrition epidemiological research. Instead, with preventative measures in place, they can conduct their work and mitigate potential ethical issues that affect themselves and their research participants. This paper emphasises the critical need for addressing ethical challenges in public health nutrition research, particularly when mental health questions are included in nutrition questionnaires. The primary ethical principles highlighted throughout the discussion include ensuring that participants fully understand the potential risks associated with participating in research, including emotional or psychological impacts from sensitive questions, which is essential. Researchers must clearly outline how sensitive topics like mental health will be handled and provide explicit details about referral procedures for those requiring clinical care. Furthermore, the handling of sensitive data, particularly related to mental health, requires the highest standards of confidentiality. It is imperative that participants’ privacy is protected at all stages of the research process. Moreover, ethical nutrition research must prioritise fairness in the recruitment and treatment of participants, especially when working with vulnerable populations. Researchers must ensure that all study designs are inclusive and considerate of the diverse needs of participants, that their findings are presented honestly without exaggeration or distortion, and that participants’ rights are safeguarded throughout the research process. Regarding participant welfare, researchers must recognise the potential psychological impact of their work, particularly when addressing sensitive issues like suicidality or depression. Ethical research must prioritise minimising harm, ensuring that participants’ mental and emotional well-being is considered in every phase of the study.

While these ethical principles are critical to nutrition research, there are several important areas for future investigation. (1) Development of standardised ethical guidelines: There is a need for standardised ethical guidelines specifically tailored to the use of mental health questionnaires in nutrition research. These guidelines should address issues such as the management of sensitive data, the process for participant referral to mental health services, and the inclusion of appropriate safeguards to prevent psychological harm. (2) Longitudinal studies on ethical challenges: Future research should examine the long-term effects of participating in nutrition studies that include mental health questions. This would help to further understand the potential psychological burden on participants and refine consent processes accordingly. (3) Training and education for researchers: Another area for future research is the development of specialised ethical training programmes for nutrition researchers. These programmes would equip researchers with the tools to navigate complex ethical dilemmas and develop protocols that prioritise both participant welfare and research integrity. (4) Exploring cultural sensitivity in ethical protocols: As nutrition research often involves diverse populations, future research should explore how ethical guidelines can be adapted to be culturally sensitive, ensuring that mental health questionnaires are appropriate and considerate for different groups.

In conclusion, this paper urges public health nutrition researchers to carefully consider ethical issues in their work, particularly when engaging with sensitive topics related to mental health. While ethical challenges are inherent in any research involving human participants, they can be mitigated through careful planning, transparency, and a commitment to the well-being of those involved. By addressing these issues head-on and fostering open discussions about ethical challenges in nutrition research, we can enhance the integrity of the field and ensure that future research continues to serve the best interests of participants and society at large.

## Data Availability

Bournemouth University aims to make our research data as openly accessible as possible. Data will be registered and discoverable via BORDaR, BU’s research data repository, and linked (where applicable) to any associated research outputs via BURO, BU’s institutional repository.

## Supplementary Materials

The following supporting information can be downloaded at: https://www.mdpi.com/article/10.3390/nu17040715/s1. File S1. A comparative analysis of previous studies’ ethical challenges and solutions, highlighting what gaps this paper aims to fill. File S2. Below are examples of sample consent forms and referral protocols as guidelines for researchers.
