# Supplementary material for: Ethical Issues and Challenges Regarding the Use of Mental Health Questionnaires in Public Health Nutrition Research
Source: Nutrients. 2025 Feb 18;17(4):715. doi: 10.3390/nu17040715 (PMC11858303; doi:10.3390/nu17040715)
Supplement: Supplementary file 1 [file nutrients-17-00715-s001.zip › nutrients-3476866-supplementary.pdf]

Supplementary File S1

Table S1. A comparative analysis of previous studies’ ethical challenges and solutions, highlighting what gaps this paper aims to fill.

	**Misclassification**	**Anonymity**	**Psychological burden**	**Researcher guilt**
**Habtamu et al. [24]**	Beck Depression Inventory-II (BDI-II) reported to be prone to misclassification.			
**McPherson et al. [15]**	Challenges associated with false positives and false negatives in depression screenings.		The psychological impact these questions may have on participants.	
**Nutting et al. [20]**				Researchers' guilt and expectations in mental health research and the emotional toll on researchers.
**Tam et al. [36]**		The inability to identify participants requiring follow-up care due to the		

		anonymous nature of many research studies.		
**Uebelacker et al. [19]**			The psychological burden of mental health questionnaires, particularly those that ask about sensitive issues like suicidality, has been widely acknowledged.	
**Gaps in previous studies**	The majority of these studies fail to provide clear recommendations on how to accurately select cut-off scores or manage misclassified participants.	While previous literature has highlighted the problem of anonymity, it often fails to address specific referral procedures for participants identified as at risk.	The solutions suggested in the literature have often been generalised and vague, such as merely advising researchers to "be mindful" of participants' emotional well-being.	Little attention has been paid to providing specific guidelines to help researchers manage these feelings.
**Our proposed solution to fill the gaps in previous literature.**	This paper not only highlights the potential risks of misclassification but also offers practical solutions, such as using a specificity threshold of	This paper expands upon this literature by proposing a comprehensive passive referral process, which ensures participants	This paper provides specific and actionable measures such as including trigger warnings, offering participants the option	This paper contributes to filling this gap by offering practical recommendations for clear communication with participants through consent forms and

	95% or higher and providing clearer guidelines for selecting cut-off scores based on specific population demographics. This paper fills a crucial gap by emphasising the need for critical evaluation of screening tools in relation to the study population, which has often been underexplored in previous research.	are provided with the necessary resources for follow-up care without compromising anonymity. By doing so, this paper offers a unique solution that has not been fully explored in the existing body of research.	to skip sensitive questions, and providing access to mental health resources. These practical strategies contribute to reducing the psychological burden on participants and ensuring the ethical integrity of the study.	information sheets. This ensures that participants have realistic expectations of the researcher's role and alleviates any unwarranted feelings of guilt on the part of the researcher.

In sum, while the existing literature on ethics in mental health research provides a solid foundation for identifying key challenges, it often lacks detailed, actionable solutions for researchers in public health nutrition and epidemiological studies. This paper fills a crucial gap by not only identifying ethical issues but also providing specific strategies and preventative measures to address these challenges, ultimately improving the ethical conduct of research in this interdisciplinary field.

Supplementary File S2:

Below are examples of sample consent forms and referral protocols as guidelines for researchers.

1. Sample Consent Form for Mental Health-Related Nutrition Research

Research Study Title: [Insert Title of Study]

Purpose of the Study
This research aims to understand the relationship between nutrition and mental health among [insert target population].

Potential Emotional or Psychological Risks
Some of the questions in this study may cause emotional discomfort or may touch on sensitive topics, including depression, anxiety, or suicidality. If you experience any distress during the survey, you are encouraged to stop at any time.

Referral Procedures

- If you disclose any distressing feelings or experiences related to depression, anxiety, or suicidal thoughts, the following procedures will be followed:
    - You will be given the contact details of mental health professionals.
    - If you wish, a trained member of the research team can directly assist in connecting you to a mental health service provider.
    - In cases where immediate intervention is needed, the researcher will contact emergency services on your behalf (only with your consent).

Voluntary Participation and Confidentiality
Your participation is entirely voluntary, and you may withdraw at any time without any consequences. All data will remain confidential and will only be used for the purpose of this research.

Contact Information
For any questions about this study or if you need emotional support, please contact:

- Principal Investigator: [Insert Contact Info]
- Emergency Mental Health Support: [Insert Contact Info]

By signing below, you indicate that you understand the purpose of this research, the potential risks, and the referral procedures.

Participant's Signature:__________
Date:__________

2. Sample Referral Protocol

Referral Protocol for Participants Reporting Mental Health Distress

1. **Initial Screening**:
   During the survey or interview, participants will be informed that questions may elicit emotional responses. Participants will be reminded that they can stop or skip any questions at any time.
2. **Identification of Mental Health Risks**:
   If participants answer certain questions in ways that suggest they may be experiencing distress (e.g., reporting feelings of depression or suicidality), researchers will:

   - Offer a follow-up brief screen for distress (e.g., the PHQ-9 or similar validated scale).
   - Assess the severity of distress and determine the appropriate referral level (urgent or non-urgent).
3. **Referral Procedure**:
   - **For Non-Urgent Cases**:
     Participants will be given a list of mental health resources, including local support hotlines and therapists, and offered assistance in contacting these resources if desired.
   - **For Urgent Cases**:
     If a participant expresses suicidal thoughts or other signs of immediate risk, the researcher will:
     - Contact emergency services with the participant's consent.
     - If consent is not given, the researcher will provide the participant with immediate contact information for local crisis helplines and encourage the participant to seek urgent care.
4. **Follow-Up**:
   The researcher will provide a follow-up check-in to ensure that the participant was able to access the necessary services, if they agree to it.
